# Respiratory uncoupling by increased H^+^ or K^+^ flux is beneficial for heart mitochondrial turnover of reactive oxygen species but not for permeability transition

**DOI:** 10.1186/1471-2121-14-40

**Published:** 2013-09-22

**Authors:** Saori Morota, Sarah Piel, Magnus J Hansson

**Affiliations:** 1 Mitochondrial Pathophysiology Unit, Lund University, Lund, Sweden; 2 Department of Anesthesiology, Tokyo Medical University, Shinjuku-ku, Tokyo, Japan; 3 Department of Clinical Physiology, Skåne University Hospital & Lund University, Lund, Sweden

**Keywords:** Ischemic preconditioning, Mitochondrial permeability transition, Potassium channels, Respiratory uncoupling, Reactive oxygen species

## Abstract

**Background:**

Ischemic preconditioning has been proposed to involve changes in mitochondrial H^+^ and K^+^ fluxes, in particular through activation of uncoupling proteins and ATP-sensitive K^+^ channels (MitoK_ATP_). The objectives of the present study were to explore how increased H^+^ and K^+^ fluxes influence heart mitochondrial physiology with regard to production and scavenging of reactive oxygen species (ROS), volume changes and resistance to calcium-induced mitochondrial permeability transition (mPT).

**Results:**

Isolated rat heart mitochondria were exposed to a wide concentration range of the protonophore CCCP or the potassium ionophore valinomycin to induce increased H^+^ and K^+^ conductance, respectively. Simultaneous monitoring of mitochondrial respiration and calcium retention capacity (CRC) demonstrated that the relative increase in respiration caused by valinomycin or CCCP correlated with a decrease in CRC, and that no level of respiratory uncoupling was associated with enhanced resistance to mPT. Mitochondria suspended in hyperosmolar buffer demonstrated a dose-dependent reduction in CRC with increasing osmolarity. However, mitochondria in hypoosmolar buffer to increase matrix volume did not display increased CRC. ROS generation was reduced by both K^+^- and H^+^-mediated respiratory uncoupling. The ability of heart mitochondria to detoxify H_2_O_2_ was substantially greater than the production rate. The H_2_O_2_ detoxification was dependent on respiratory substrates and was dramatically decreased following calcium-induced mPT, but was unaffected by uncoupling via increased K^+^ and H^+^ conductance.

**Conclusion:**

It is concluded that respiratory uncoupling is not directly beneficial to rat heart mitochondrial resistance to calcium overload irrespective of whether H^+^ or K^+^ conductance is increased. The negative effects of respiratory uncoupling thus probably outweigh the reduction in ROS generation and a potential positive effect by increased matrix volume, resulting in a net sensitization of heart mitochondria to mPT activation.

## Background

Hearts exposed to brief periods of ischemia exhibit considerable protection from subsequent ischemia reperfusion-injury, so-called ischemic preconditioning [1,2]. Activation of mitochondrial permeability transition (mPT) has been demonstrated to play a critical role in several animal models of disease including ischemia-reperfusion injury, and inhibition of the mPT regulator cyclophilin D is cardioprotective in animal models and in man [3-6]. The mechanisms involved in preconditioning are not fully established but inhibition of mPT is generally regarded as an important end effect [7,8].

Changes in mitochondrial H^+^ or K^+^ conductance may critically influence mitochondrial function. Several different pathways involving altered H^+^ or K^+^ entry into the mitochondrial matrix have been proposed to modify resistance to ischemia-reperfusion injury. Mitochondrial K^+^ channel activation, in particular the putative ATP-sensitive K^+^ channel (MitoK_ATP_), has been extensively investigated as a mediator of ischemic preconditioning [9]. Uncoupling proteins - *i.e.* mitochondrial transporter proteins structurally related to UCP1, which uncouples mitochondrial respiration in brown adipose tissue - have also been explored as mediators of ischemic preconditioning [10]. Activation of K^+^ channels and H^+^ translocators will both uncouple mitochondrial oxidative phosphorylation to some extent. So-called mild uncoupling [11] has been suggested to reduce the sensitivity of mitochondria to undergo calcium-induced mPT both by reducing the ∆Ψ_m_-dependent calcium uptake and by attenuating reactive oxygen species (ROS) production of mitochondria [12,13]. Low concentrations of protonophores can induce a ROS-dependent cardioprotection in ischemia-reperfusion injury [14]. Similarly, activation of mitochondrial K^+^ channels has been proposed to reduce the generation of ROS [15,16]. However, results are not consistent as the opposite relation also has been found with an increase in mitochondrial ROS production following K^+^ channel activation [17]. A signaling pathway linking mitoK_ATP_ and mPT has also been proposed where an increased generation of H_2_O_2_ activates an mPT-associated PKCϵ [18,19]. The increased K^+^ conductance following mitochondrial K^+^ channel activation may also increase matrix volume [20]. We have previously demonstrated that enhanced matrix volume in brain mitochondria increased their resistance to calcium-induced mPT [21].

The objective of the present study was to explore how increased H^+^ and K^+^ conductance influence a number of heart mitochondrial physiological parameters relevant for ischemia-reperfusion injury, including respiration, calcium retention capacity (CRC), matrix volume, ROS production and ROS scavenging. Further, we wanted to explore whether increased matrix volume in heart mitochondria exert a beneficial effect on CRC.

In summary, we demonstrate that decreased matrix volume reduces mitochondrial resistance to calcium-induced mPT whereas increased volume did not alter it. H_2_O_2_ generation of mitochondria was reduced by both K^+^- and H^+^-mediated respiratory uncoupling whereas scavenging of H_2_O_2_, which was substantially greater than the production rate, was unaffected by uncoupling unless the inner mitochondrial membrane integrity was disrupted. Increased H^+^ or K^+^ flux dose-dependently decreased mitochondrial calcium retention capacity and no level of uncoupling demonstrated any positive effect on heart mitochondrial resistance to calcium-induced mPT.

## Results

### Matrix volume

**Figure 1:**
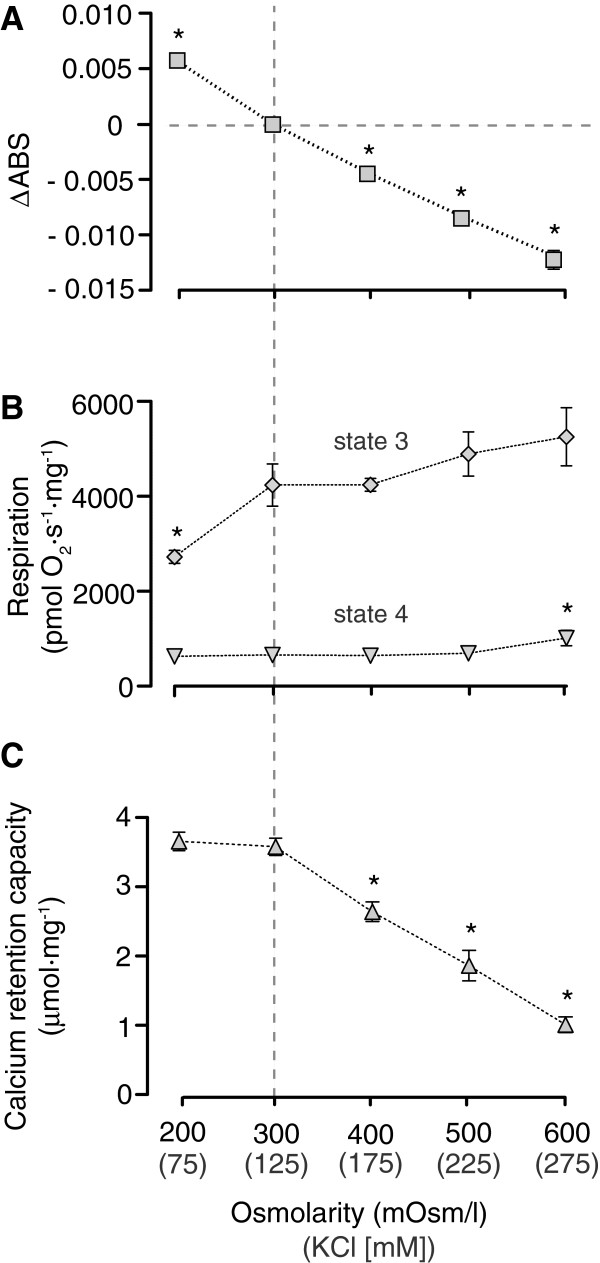
**Matrix volume effects on heart mitochondrial respiration and calcium retention capacity (CRC).** Heart mitochondrial matrix volume was modulated by changing the osmolarity of the buffer using different concentrations of KCl. The KCl concentrations and the respective approximate medium osmolarities are depicted on the x-axis. The standard incubation medium, 125 mM KCl, is marked by vertical dashed line **(A)** Changes in matrix volume were monitored by assaying changes in light absorbance at 520 nm (∆ABS). **(B)** Respiration rates at state 3 (during ADP phosphorylation) and state 4 (after ADP was converted to ATP) are demonstrated. **(C)** Calculations of mitochondrial CRC at the different buffer osmolarities. Values are means ± S.E.M. * indicates p < 0.05 compared to control (125 mM KCl).

Activation of mitochondrial K^+^ channels with influx of K^+^ along its electrochemical gradient may lead to different extents of matrix volume increase depending on the balance of K^+^ entry versus activity of the K^+^/H^+^ exchanger. Here we explored the relation between heart mitochondrial matrix volume and the mitochondrial resistance to calcium-induced mPT by infusing calcium to mitochondria suspended in media with different osmolarities. The extent of mitochondrial calcium accumulation before activation of permeability transition, the calcium retention capacity (CRC), is plotted against the concentration of KCl and the resulting approximate medium osmolarity in Figure 1C. Changes in mitochondrial light absorbance are depicted in Figure 1A, and respiratory function was evaluated in parallel experiments (Figure 1B). CRC was dose-dependently decreased in relation to medium osmolarity in hyperosmolar buffer, compared to the standard buffer containing 125 mM KCl (300 mOsm/l). Mitochondria with reduced matrix volume were thus more sensitive to calcium-induced mPT. The reduced matrix volume tended to increase respiration with a significant increase in state 4 respiration at 275 mM KCl but no significant effect on state 3 respiration. In contrast, increasing mitochondrial matrix volume by reducing the KCl concentration and osmolarity of the media from 125 mM to 75 mM had no apparent effect on CRC but state 3 respiration and the corresponding respiratory control ratio (RCR) were reduced. Exchanging KCl for LiCl did not alter the osmolarity effect; there was no difference in CRC between mitochondria suspended in 50 mM LiCl plus 125 mM KCl buffer and mitochondria suspended in 175 mM KCl buffer. Increasing osmolarity 200 mOsm/l using sucrose instead of KCl likewise decreased CRC (data not shown). In summary, matrix volume correlated to mitochondrial resistance to calcium-induced mPT as reduced volume dose-dependently reduced CRC. However, increased matrix volume failed to increase heart mitochondrial resistance to mPT and negatively affected respiratory function.

### Uncoupling and resistance to mPT

**Figure 2:**
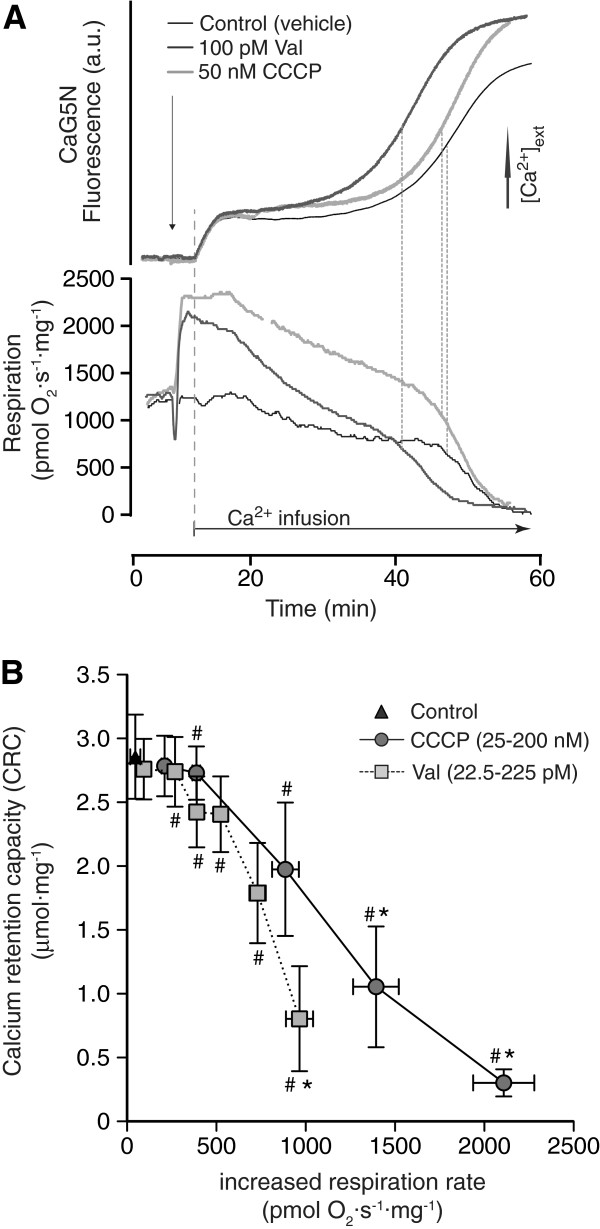
**Effects of respiratory uncoupling via increased K**^**+ **^**or H**^**+ **^**conductance on heart mitochondrial calcium retention. (A)** Representative traces of simultaneous measurements of extramitochondrial calcium concentration, assessed by Calcium green 5 N (CaG5N) fluorescence (a.u. = arbitrary units), and mitochondrial respiration rates before and during calcium infusion. Both the K^+^ ionophore valinomycin (Val) and the protonophore CCCP at indicated concentrations induced increased basal respiration rates following their additions. Infusion of calcium, 160 nmol·min^-1^·mg^-1^, induced a rise in extramitochondrial calcium concentration until a set-point where mitochondria accumulated calcium at the same rate as that infused. Calcium accumulation of mitochondria was followed by calcium release and respiratory inhibition attributed to activation of permeability transition throughout the mitochondrial population (mPT). Vertical dashed lines to the right indicate start of maximal calcium release and the corresponding rapid phase of respiratory inhibition. **(B)** Calcium retention capacity (CRC) of mitochondria was calculated as the amount of infused calcium until start of the rapid phase of respiratory inhibition. The CRCs are plotted against the increases in basal respiration rates caused by the addition of 22.5-225 pM valinomycin or 25–200 nM CCCP in the respective experiments. Values are means ± S.E.M. * and # indicate p < 0.05 compared to control for CRC and respiration respectively.

Increased H^+^ or K^+^ conductance will lead to an augmented uncoupling of mitochondrial respiration since the proton motive force is utilized for ion exchange rather than ATP synthesis. In order to evaluate if uncoupling can reduce the sensitivity of heart mitochondria to undergo calcium-induced mPT we performed a detailed simultaneous analysis of mitochondrial CRC and respiration (Figure 2). Mitochondria were exposed to wide concentration ranges of the K^+^ carrier valinomycin, (22.5 to 225 pM) and the protonophore CCCP (25 to 200 nM). The uncoupling effects of valinomycin or CCCP were calculated by measuring the added rates of respiration following their addition. Representative traces of a control and two of the lower concentrations of the respective compounds are demonstrated in Figure 2A. When mPT is activated throughout the mitochondrial population, respiration is decreased at least partly due to a loss of NADH/NAD^+^ from the mitochondrial matrix [21], and the decrease in respiration occurs simultaneously to the rapid release of calcium from mitochondria following the period of calcium uptake (Figure 2A). Plotting mitochondrial CRC against the increased respiration rate demonstrated a dose-dependent reduction in CRC for both the K^+^ carrier and the protonophore (Figure 2B). Linear regression analysis of the individual CCCP and valinomycin data points demonstrated a significant correlation between increases in basal respiratory rates and decreased CRC. The r^2^ values were 0.76 (p < 0.0001) and 0.45 (p = 0.0001) for CCCP and valinomycin, respectively. At higher concentrations of the two compounds, valinomycin caused a more pronounced reduction of CRC compared to CCCP at comparable degrees of uncoupling. In summary, no level of respiratory uncoupling demonstrated any positive effect on heart mitochondrial resistance to calcium-induced mPT.

### Effects of diazoxide and cyclosporin A

The putative activator of MitoK_ATP_, diazoxide, was evaluated for effects on respiration and CRC (in separate experiments) at 30, 100 and 200 μM. State 4 respiration was slightly but significantly elevated at 100 and 200 μM diazoxide (862.2 ± 44 and 937.1 ± 33, respectively, compared to 659.2 ± 30 pmol O_2_∙s^-1^∙mg^-1^ for control). The corresponding respiratory control ratios (state3/state4) were reduced but none of the tested concentrations of diazoxide had any significant effect on CRC (data not shown). The cyclophilin D inhibitor CsA (1 μM), in contrast, significantly increased CRC from 3.5 ± 0.4 to 4.7 ± 0.2 μmol∙mg^-1^.

### ROS generation

**Figure 3:**
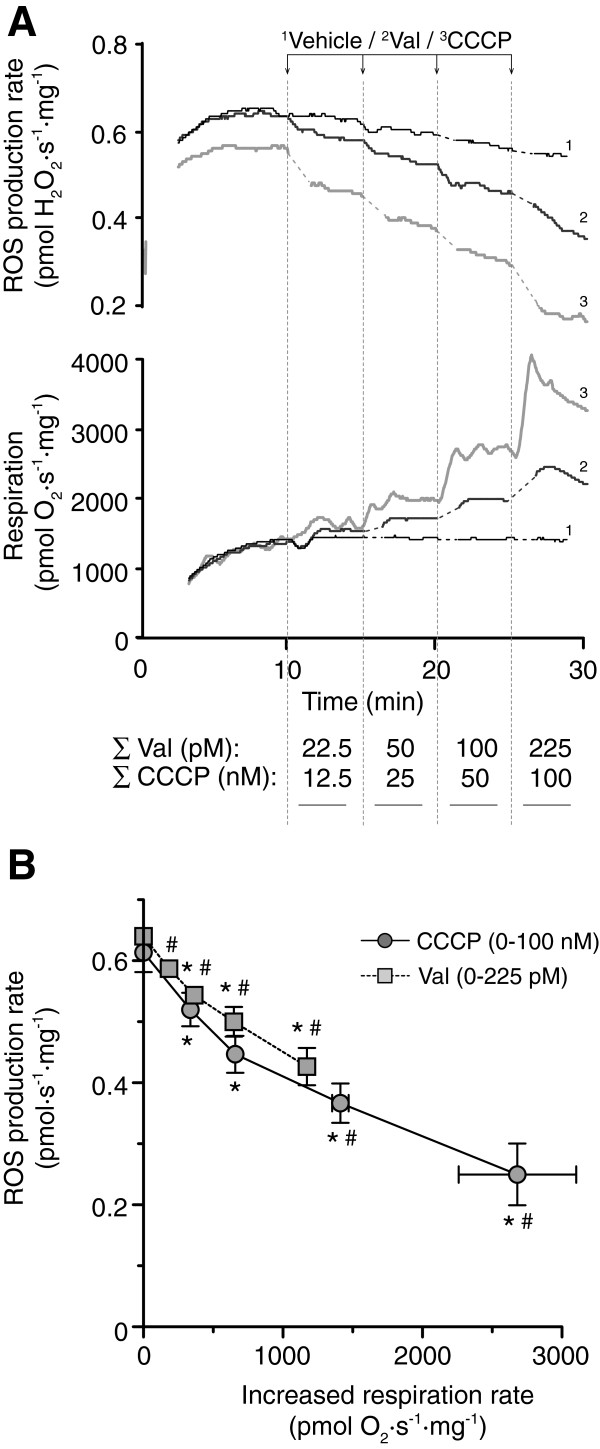
**Mitochondrial ROS generation following uncoupling by K**^**+ **^**ionophore or protonophore. (A)** Representative traces of simultaneous measurements of mitochondrial H_2_O_2_ production rates, assessed by following the oxidation of Amplex Red to the fluorescent product resorufin, and respiration rates following sequential additions of vehicle (ethanol, control), valinomycin (Val) and CCCP. The resulting concentrations of Val and CCCP are depicted below the x-axis at the respective time intervals. **(B)** The quantitative effects of increasing K^+^ and H^+^ conductances on ROS generation are illustrated by plotting the rates of mitochondrial H_2_O_2_ production against the increased respiration rates for the respective concentrations of Val and CCCP. Values are means ± S.E.M. * and # indicate p < 0.05 compared to the initial value with no uncoupling agent present for H_2_O_2_ production rate and respiration respectively.

To evaluate whether there is a qualitative or quantitative difference between increased H^+^ and K^+^ conductance on ROS generation, heart mitochondria were exposed to repetitive additions of valinomycin (22.5 to 225 pM) and CCCP (25 to 100 nM). The H_2_O_2_ production rates were correlated to simultaneous measurements of increased respiration rates. The K^+^ carrier and the protonophore dose-dependently reduced mitochondrial ROS generation corresponding to extent of respiratory uncoupling (Figure 3). Linear regression analysis of the individual CCCP and valinomycin data points demonstrated a significant correlation between increases in basal respiratory rates and decreased H_2_O_2_ production. The r^2^ values were 0.65 (p = 0.0003) and 0.85 (p < 0.0001) for CCCP and valinomycin, respectively. There was no significant difference between the slopes for CCCP or valinomycin. ROS generation was also evaluated in mitochondria exposed to a continuous calcium infusion in presence of 50 pM valinomycin or 100 nM CCCP which both reduced H_2_O_2_ detection from 4.3 ± 0.2 to 3.77 ± 0.1 and 3.3 ± 0.1 pmol∙s^-1^∙mg^-1^, respectively. In summary, there was no qualitative or quantitative difference on the effect on heart mitochondrial H_2_O_2_ generation between increased H^+^ and K^+^ conductance.

### ROS scavenging

**Figure 4:**
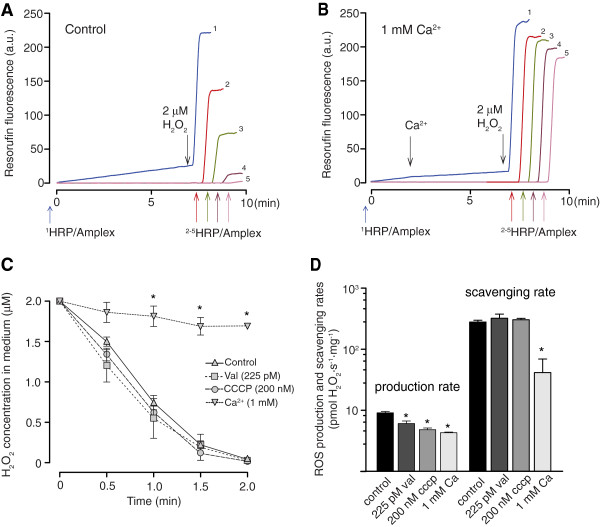
**Mitochondrial ROS scavenging ability following uncoupling or permeability transition. (A)** Representative traces of ROS scavenging ability measurements. In trace 1, SOD, HRP and Amplex Red were added from start and 2 μM H_2_O_2_ was added at 7 min to obtain a reference value*.* In trace 2–5, HRP and Amplex Red were added 0.5, 1, 1.5 and 2 min after H_2_O_2_ administration, respectively, to measure the residual H_2_O_2_ concentrations and hence determine the mitochondrial ROS scavenging ability. **(B)** Same as panel A but with 1 mM Ca^2+^ present to induce permeability transition. **(C)** Mean residual H_2_O_2_ concentrations in the mitochondrial suspensions following addition of 2 μM H_2_O_2_. Experiments were performed with control, 225 pM valinomycin (Val), 200 nM CCCP and calcium-treated mitochondria. **(D)** Comparison of mitochondrial ROS production and ROS scavenging rates in heart mitochondria. Note the logarithmic scale. ROS scavenging rate following H_2_O_2_ addition was calculated over the first minute following addition of H_2_O_2_ and ROS production rate was calculated in the same set of experiments before addition of exogenous H_2_O_2_ for those samples where the Amplex Red system was present from start. Values are means ± S.E.M.* indicates p < 0.05 compared to control.

A reduced capacity of mitochondria to detoxify ROS could potentially be more important for organ failure than gross mitochondrial ROS generation. The steady-state production of ROS is generally assessed by probes with higher affinity for H_2_O_2_ than the endogenous mitochondrial scavenging pathways, and the balance between mitochondrial generation and detoxification of H_2_O_2_ is therefore not reflected by such an assay [22,23]. Further, there is no accumulation of H_2_O_2_ in suspensions of isolated intact mitochondria in contrast to mitochondria following mPT [22-24]. Therefore, to calculate the rate of H_2_O_2_ removal, the heart mitochondrial suspensions were exposed to exogenous H_2_O_2_ and the ROS detection system was added after specific time points to determine residual H_2_O_2_ concentrations (Figure 4). Following a large bolus load of calcium to induce extensive mPT (Figure 4B) or when the experiments were performed without respiratory substrates (data not shown) the H_2_O_2_ removal was dramatically diminished, and the exogenously added H_2_O_2_ remained in the mitochondrial suspension to a large extent. However, valinomycin and CCCP had no effect on H_2_O_2_ removal (Figure 4C) when tested at concentrations that displayed prominent effects on respiration and CRC. When compared in the same set of experiments under standard conditions, the mitochondrial H_2_O_2_ scavenging rate was more than 10-fold greater than the detected production rate of H_2_O_2_ (Figure 4D). Thus, the ROS scavenging of heart mitochondria was dependent on active substrate oxidation and required an intact inner membrane. Whereas uncoupling by increased H^+^ and K^+^ conductance reduced ROS production, it did not affect ROS scavenging.

## Discussion

Several hypotheses on the mechanisms of ischemic preconditioning involve activation of channels or transporter proteins affecting the ion flux, most notably for K^+^ and H^+^, across the inner mitochondrial membrane. Increasing mitochondrial K^+^ or H^+^ conductance will have several physiological consequences. The aim of the present work was to explore how some of these alterations relate to commonly proposed mechanisms for ischemia-reperfusion injury in heart mitochondria.

An extensive uncoupling will cause a dramatic effect on normal mitochondrial function, but so-called mild uncoupling has been suggested to reduce the sensitivity of mitochondria to undergo calcium-induced mPT by limiting the calcium uptake and ROS production of mitochondria [12,25]. The CRC assay is a sensitive and quantitative method for evaluating mitochondrial susceptibility to mPT induction [26], and by simultaneous monitoring of respiration the specificity is increased [21]. Here, we demonstrate that heart mitochondrial CRC is dose-dependently reduced by uncoupling irrespective of whether H^+^ or K^+^ conductance is increased, and that the reduced CRC is caused by mPT (as demonstrated by the respiratory decrease). There was no tendency of any beneficial effect at lower concentrations of the K^+^ carrier valinomycin or the protonophore CCCP. At the highest tested concentration of CCCP, 200 nM, the respiratory increase was less than a state 2 to state 3 transition but the ability to retain Ca^2+^ was virtually abolished and mitochondria underwent permeability transition at a very low calcium load. Ca^2+^ is the principal trigger of mPT and a reduction in matrix Ca^2+^ would arguably reduce mPT activation. However, it is the free Ca^2+^ ions that trigger mPT and most calcium in mitochondria is retained as inactive calcium-phosphate complexes [26]. The formation of calcium-phosphates is highly dependent on an alkaline matrix environment. A small decrease in ∆pH, as occurs with uncoupling following H^+^ entry, will increase the concentration of free Ca^2+^ even if the electrophoretic driving force for Ca^2+^ uptake is reduced. Uncoupling may also directly increase the propensity of the mPT pore to open due to a more oxidized redox state and a reduced mitochondrial membrane potential (∆Ψ_m_) [27,28].

Increasing K^+^ flux into the mitochondria may affect several factors important for calcium handling and mPT regulation, including ∆Ψ_m_, ∆pH, ROS generation and matrix volume, which are somewhat different to increasing H^+^ flux. For example, increasing K^+^ or H^+^ conductance may be expected to have opposite effects on ∆pH due to activation of the K^+^/H^+^ exchanger by the former. Whether an alteration in any of these parameters is beneficial or not to the resistance of mitochondria to the dramatic consequences of calcium overload depend both on their direct and potential indirect effects on the mPT pore components, as pointed out above for the effect of ∆pH on free Ca^2+^. In a previous study, we found that low increases in K^+^ but not H^+^ conductance was beneficial to brain mitochondrial resistance to calcium-induced permeability transition, and that the beneficial effect probably was mediated by an increase in matrix volume [21]. In the present study, there was no qualitative difference between increasing H^+^ or K^+^ conductance on CRC. The negative effects of uncoupling thus predominate over other potential direct beneficial effects of increasing K^+^ conductance in rat heart mitochondria.

Changes in mitochondrial matrix volume influence respiratory function [29-31] and we have previously demonstrated that increased matrix volume in brain mitochondria either caused by an elevated influx of K^+^ or by suspending mitochondria in hypoosmolar KCl medium increases CRC [21]. In the present study we explored respiratory changes and CRC in heart mitochondria suspended in hypo- or hyperosmolar KCl media. Increasing medium osmolarity by KCl (or LiCl) dose-dependently decreased CRC indicating a positive correlation between matrix volume and CRC. A possible confounder to increasing osmolarity with KCl or LiCl is the simultaneous change in ionic strength which may affect *e.g.* cytochrome *c* charge interactions [32]. However, increasing medium osmolarity with sucrose likewise decreased CRC. In contrast to the effects of increasing medium osmolarity, decreasing the osmolarity further than the standard conditions did not cause the expected increase in CRC. A plausible explanation may be the reduced respiratory capacity of heart mitochondria in the hypoosmolar medium. An increase in matrix volume has been shown to cause release of cytochrome *c* thus reducing respiratory capacity in isolated liver mitochondria [33]. The same authors also found a reduced calcium uptake in mitochondria following exposure to a hypoosmotic shock. In a limited set of experiments in heart mitochondria (data not shown) we found that there was a tendency for exogenously added cytochrome *c* to increase respiration more in hypoosmolar media compared to hyperosmolar conditions, but these findings were not explored further. It is thus plausible that any beneficial effect on heart mitochondrial CRC by an enlarged matrix volume following increased K^+^ flux is counteracted by a respiratory inhibition and a direct negative effect of uncoupling. Even though the majority of studied characteristics described herein were similar to previous findings in brain mitochondria, there seems to be a distinct organ specific difference in relation to the response to increased K^+^ conductance and matrix volume.

ROS production of mitochondria has since long been recognized to increase with elevated proton-motive force [34,35]. This is particularly evident when isolated mitochondria are supplemented with solely succinate as respiratory substrate to support so-called reverse electron transport through respiratory complex I [36]. Ischemic preconditioning reduces oxidative damage in mitochondria, and a reduction of oxidative stress may mediate a reduced propensity for mPT activation [37]. Previous studies have demonstrated both increased and decreased ROS generation following augmented K^+^ flux in mitochondria [15-19]. In the present study, both increased K^+^ and H^+^ flux dose-dependently reduced the production of H_2_O_2_ in heart mitochondria using complex I substrates. There was no apparent qualitative or quantitative difference between increased K^+^ and H^+^ flux; rather, the level of ROS reduction was in proportion to the extent of uncoupling. However, the reduced ROS generation did not translate into an increased resistance to mPT induction.

The capacity of mitochondria to detoxify ROS is less explored but likely equally or more important than mitochondrial ROS production in oxidative stress [23] and there is no clear evidence whether mitochondria function as net source or sink of ROS [38]. Heart mitochondria are equipped with effective antioxidant systems for scavenging of ROS and dysfunction of these are involved in cardiovascular disease [39]. Malfunction of mitochondrial thioredoxin reductase causes dilated cardiomyopathy in man [40] and aggravates ischemia-reperfusion injury in mice [41], while overexpression of glutaredoxin-2 reduces ischemia-reperfusion injury in mice [42]. The mitochondrial antioxidative systems are dependent on intact respiratory function. Reduced glutathione and NADPH provide antioxidant defences against H_2_O_2_ mediated damage through the action of *e.g.* the glutathione peroxidase family, peroxiredoxins and glutaredoxins. The reduction of GSH and thioredoxin reductase is dependent on NADPH generation from *e.g.* the proton motive force-driven transhydrogenase [43]. Although uncoupling of mitochondrial respiration reduces ROS production, any interference with ROS scavenging may result in an overall negative effect on mitochondrial ROS handling. Therefore, we investigated the heart mitochondrial capacity of H_2_O_2_ removal under conditions of uncoupling and calcium overload. The mitochondrial H_2_O_2_ scavenging rate was more than 10-fold greater than the detected production rate of H_2_O_2_ under standard conditions. Whereas uncoupling decreased H_2_O_2_ production it did not affect H_2_O_2_ scavenging. In contrast, calcium overload leading to mPT dramatically reduced H_2_O_2_ scavenging in line with previous reports [23,24]. Any effects of calcium unrelated to mPT activation were not explored in the present study.

We did not detect any specific effect of the MitoK_ATP_ opener diazoxide. The compound uncoupled respiration at high concentrations, which also has been demonstrated previously [20]. The present experiments were not primarily designed to explore the putative MitoK_ATP_. Such experiments are usually performed within the first few minutes upon incubation in experimental medium and the present experiments examining general effects of increased H^+^ and K^+^ conductance required a longer time frame. It has been noted that the MitoK_ATP_ is sensitive to rapid inactivation [44] and there is also a current debate over its existence [9].

## Conclusions

The present findings suggest that there are no direct beneficial effects of increased K^+^ or H^+^ flux on heart mitochondrial resistance to mPT. Even though there was a decrease in the generation of ROS and a positive correlation between matrix volume and CRC in hyperosmotic media, the negative effects related to respiratory uncoupling predominated with a net sensitization of heart mitochondria to mPT activation. Importantly though, uncoupling did not hamper mitochondrial ROS scavenging capacity. It is concluded that respiratory uncoupling is not beneficial to rat heart mitochondrial resistance to calcium overload regardless of whether H^+^ or K^+^ conductance is increased. The present study is limited by its specific examination of mitochondria in their isolated state in contrast to their integrated function *in vivo*. Nevertheless, the results imply that the direct physiological effects of increased H^+^ or K^+^ flux on ROS generation, matrix volume and calcium handling in heart mitochondria are not in themselves sufficient to mediate ischemic preconditioning in the heart.

## Methods

### Isolation of heart mitochondria

Adult male Wistar rats (Harlan Scandinavia ApS, Allerød, Denmark, 350–450, 500–600 g) were allowed access to water and food *ad libitum* prior to use. All animal procedures were approved by the Malmö/Lund Ethical Committee for Animal Research (M44-07, M99-10). Following a brief exposure to halothane in order to minimize stress, animals were decapitated [45]. Hearts were rapidly transferred into ice-cold solution and mitochondria were isolated using trypsinization, homogenization and differential centrifugation at 4°C as previously described [46]. Protein content was measured using Bradford analysis.

### Calcium retention capacity (CRC), mitochondrial respiration and matrix volume changes

A luminescence spectrometer LS-50B (Perkin-Elmer, Emeryville, CA) with a temperature controlled cuvette holder or an Oxygraph-2 k with a Titration-Injection micro Pump TIP-2 k (Oroboros instruments, Innsbruck Austria) was used to measure mitochondrial CRC. Experiments were performed at 37°C. Mitochondria, 75 μg/ml, were suspended in buffer containing 125 mM KCl, 20 mM Trizma base, 2 mM Pi (K_2_PO_4_), 1 mM MgCl_2_, 1 μM EGTA, 200 μM ATP, 10 μM BSA and 5 mM of the NADH-linked respiratory substrates malate and glutamate, pH 7.1. For experiments assessing matrix volume effects, the osmolarity of the buffer was altered by using different KCl concentrations, 75–275 mM, corresponding to approximately 200–600 mOsm/l. In experiments evaluating modulation of the putative MitoK_ATP_, 30, 100 or 200 μM diazoxide was added before addition of mitochondria. For evaluation of cyclophilin D modulation of mPT, 1 μM CsA was used. The mitochondrial suspensions were infused with 160 nmol CaCl_2_∙min^-1^∙mg^-1^ in presence of 1 μg/ml oligomycin and 50 μM ADP. In luminescence spectrometer experiments, mitochondrial Ca^2+^ uptake/release was monitored by the excitation ratio of the extramitochondrial calcium-sensitive fluorescent probe Fura 6 F (250 nM, Ex. 340/380 nm, Em. 509 nm). In experiments monitoring oxygen consumption, mitochondrial Ca^2+^ uptake and release was monitored by respiration changes during Ca^2+^ infusion [21]. In one set of experiments, respiration and extramitochondrial calcium were monitored simultaneously by following the fluorescence of Calcium Green 5 N (100 nM) in the oxygraph using a O2k-Fluorescence LED2-Module (Oroboros instruments, Innsbruck Austria).

CRC was calculated as the amount of infused calcium from the start of mitochondrial calcium uptake until start of maximal calcium release or start of the rapid phase of respiratory inhibition. The effects of changing medium osmolarities on respiratory states were investigated under conditions described above except that no adenine nucleotides were present from start. State 3 respiration was induced by adding 250 μM ADP and state 4 respiration was measured after all ADP had been converted to ATP. Matrix volume changes were evaluated by measuring light absorbance at 520 nm in a Hitachi U-2800 Spectrophotometer (Tokyo, Japan).

### Mitochondrial ROS production and scavenging

Mitochondrial H_2_O_2_ generation was detected by the oxidation of 1 μM Amplex Red to the fluorescent product resorufin in presence of HRP and SOD (0.5 and 20 U/ml, respectively) [24]. Experiments were performed under the same conditions as described above. Simultaneous measurement of respiration and H_2_O_2_ generation was performed in the oxygraph [47]. The oxidation of Amplex Red was calibrated with repetitive additions of 0.1 μM H_2_O_2_, and the calibrated H_2_O_2_ concentrations and production rates were monitored. Mitochondrial ROS scavenging experiments were performed in the luminescence spectrometer. In contrast to following the continuous Amplex Red oxidation, HRP and Amplex Red were added at specific time points (0.5 – 2.0 min) following addition of 2 μM H_2_O_2_ to the mitochondrial suspensions in order to detect the remaining H_2_O_2_ concentration and to establish the rate of mitochondrial H_2_O_2_ detoxification.

### Statistical analyses

All average results are presented as mean ± S.E.M. and were evaluated using one-way ANOVA or repeated measures ANOVA for comparisons within experimental runs followed by Dunnett’s multiple comparison post hoc test using GraphPad Prism v5.0 software. All experiments were replicated at least 3 times in separate mitochondrial preparations. Differences were considered significant where *P* < 0.05.

## Competing interests

MJH has equity interest and receives consultancy fees and SP is a part-time employee of NeuroVive Pharmaceutical AB, which is active in the field of mitochondrial medicine including development of cyclophilin D inhibitors.

## Authors’ contributions

SM and SP performed experiments. SM and MJH planned and designed the experiments, analysed the results and wrote the manuscript. All authors read and approved the final manuscript.
